# Comparison of Thermoresponsive Hydrogels Synthesized by Conventional Free Radical and RAFT Polymerization

**DOI:** 10.3390/ma12172697

**Published:** 2019-08-23

**Authors:** Fanny Joubert, Peyton Cheong Phey Denn, Yujie Guo, George Pasparakis

**Affiliations:** UCL School of Pharmacy, 29-39 Brunswick Square, London WC1N 1AX, UK

**Keywords:** RAFT polymerization, PNIPAAm gels, free radical polymerization, thermoresponsive hydrogels

## Abstract

We compared the influence of the polymerization mechanism onto the physical characteristics of thermoresponsive hydrogels. The Poly(*N*-isopropylacrylamide) (PNIPAAm) hydrogels were successfully synthesized using reversible addition-fragmentation chain-transfer (RAFT) and free radical polymerization (FRP). The gels were prepared while using different crosslinker feed and monomer concentration. The swelling, dye release, and hydrolytic stability of the gels were investigated in water, or in representative komostrope and chaotrope salt solutions at room temperature and at 37 °C. It was found that the swelling ratio (SR) of the RAFT gels was significantly higher than that of the FRP gels; however, an increased crosslinking density resulted in a decrease of the SR of the RAFT gels as compared to the corresponding gels that are made by FRP, which indicates the limitation of the cross-linking efficiency that is attained in RAFT polymerization. Additionally, an increased monomer concentration decreased the SR of the RAFT gels, whereas a similar SR was observed for the FRP gels. However, the SR of both RAFT and FRP gels in NaSCN and Na_2_SO_4_ solutions were similar. Finally, the rate of dye release was significantly slower from the RAFT gels than the FRP gels and the hydrolytic stability of the RAFT gels was lower than that of FRP gels in water, but maintained similar stability in Na_2_SO_4_ and NaSCN solutions.

## 1. Introduction

Over the last decades, controlled polymerization methods have literally transformed the field of polymer science and they have enabled polymeric materials to be facilely integrated with the biomedical sciences finding numerous applications in various contexts, as biomaterials, sensing platforms, and drug delivery systems, among others [1,2,3].

Arguably the most widely used reversible-deactivation polymerizations (RDRPs) include nitroxide-mediated polymerization (NMP) [4,5,6], atom transfer radial polymerization (ATRP) [7,8,9,10,11,12,13], and reversible addition-fragmentation chain transfer (RAFT) polymerization [14,15,16,17,18]. These methods allow for unprecedented control on the molecular structure of the final polymer and confer synthetic fidelity and reproducibility that was inaccessible with traditional polymerization methods (i.e., free radical polymerization, FRP). This leads to a better understanding of structure-to-function properties and it also helps us to design intricate polymer structures of complex molecular topology. More particularly, RAFT polymerization [18,19,20] has been used to copolymerize (di-)vinyl monomers to form more homogeneous and structurally well-defined three-dimensional (3D) networks that are difficult, if not impossible, to obtain via the conventional FRP route [19,21]; these networks find numerous applications as swelling matrices, cell/drug encapsulants, separation technologies, self-healing materials, and responsive sensors and actuators. Recently, several groups reported on the macroscopic differences between RAFT and FRP-made polymer networks [21,22,23,24,25,26]; it is generally accepted that FRP networks tend to produce less well-defined polymer meshes with collapsed micro-domains, which in turn compromise the (de-)swelling or responsive properties of the bulk materials macroscopically. Conversely, RAFT produced gels with narrow polymer mesh distributions with very good control on the polymerization kinetics.

Poly(*N*-isopropylacrylamide) (PNIPAAm) is the gold standard polymer in the field of responsive polymers, owing to its lower critical solution temperature (LCST) that is close to body temperature, and its ease of preparation by virtually all radical polymerization methods [27,28]. PNIPAAm polymers have found applications in many different formats as soluble or self-assembled constructs, as surface coatings, and in the form of responsive hydrogels [3,29]. The LCST in aqueous media is known to be highly dependent on salt type and concentration [30], owing to the fine balance of the hydrophobic domains of the polymers (these are the polymer backbone and the isopropyl segments) and the hydrogen bond forming amide moieties. Although the effect of the so-called chaotrope and kosmotrope salts on the LCST is relatively well-established, their effect on the volume phase transition temperature (VPTT) in three-dimensional polymer networks with respect to the polymerization route is less explored.

In this report, we compare the physicochemical properties of two groups of gels, which were synthesized by FRP and RAFT polymerization. It is demonstrated that the gels significantly differ in key properties such as the swelling ratio in different chao-/kosmo-tropic solutions, their release rates of model dyes, and in their chemical stability. We attribute these large differences in the refinement of the molecular fidelity that RAFT confers on the polymer network, which collectively leads to marked differences in the macroscopic properties as compared to the gels that are synthesized by FRP.

## 2. Materials and Methods

### 2.1. Materials

*N*-isopropylacrylamide (NIPAAm), 2,2-azobisisobutyronitrile (AIBN), 1,4-dioxane, hexane, acetone, 2-(dodecylthiocarbonothioylthio)-2-methylpropionic acid, *N*,*N′*-methylenebis(acrylamide), fluorescein *O*-methacrylate, fluorescein (free acid), sodium thiocyanate (NaSCN), and sodium sulfate (Na_2_SO_4_) were purchased from Sigma Aldrich (St. Louis, MO, USA). Deuterated chloroform was supplied from Cambridge Isotope Ltd., (Tewksbury, MA, USA) and *N*,*N*-dimethylformamide (DMF) was purchased from Rathburn (Walkerburn, UK).

### 2.2. Characterizations

Solution state NMR was performed while using a Bruker Avance 400 spectrometer (Coventry, UK). Size exclusion chromatography (SEC) was conducted with DMF as the mobile phase containing 5 mM NH_4_BF_4_ additive at 70 °C with a flow rate of 1.00 mL/min. 100 µL of polymer aliquots in DMF (5 mg/mL) were injected in a Viscotek system that was equipped with a refractive index (RI) detector. Poly(methyl methacrylate) (PMMA) standards were used for calibration and OMNISEC software (version 5.1) was used to determine the average molecular weight (*M*_n_) and dispersity (Đ_M_). A Cary 100 UV-vis spectrophotometer from Agilent Technologies (Santa Clara, CA, USA) was used to record UV-vis absorption spectra. A SpectraMax^®^ M2e multimode microplate reader (Molecular Devices, San Jose, CA, USA) was used to measure the absorbance of the samples. A FEI Quanta 200 FEG SEM (Waltham, MA, USA) was used to record images of the hydrogels.

### 2.3. Experimental

#### 2.3.1. Free Radical Polymerization (FRP) of NIPAAm

In a 25 mL one-neck round-bottom flask, NIPAAm (1 g, 8.837 mmol) and AIBN (1.45 mg, 0.009 mmol) were dissolved in dioxane (10 mL). The flask was sealed with a rubber septum and then purged while using argon for 15 min. The flask was heated at 60 °C for 16 h under magnetic stirring. The reaction was stopped by exposing the solution in open air and the polymer/monomer mixture was precipitated in hexane (100 mL). The polymer F1 was obtained as a white powder with a yield of 82.5% (0.82 g) and it was characterized using NMR spectroscopy and SEC.

#### 2.3.2. RAFT Polymerization of NIPAAm

In a 25 mL one-neck round-bottom flask, NIPAAm (1 g, 8.837 mmol, 100 eq.), 2-(dodecylthiocarbonothioylthio)-2-methylpropionic acid (32.22 mg, 0.088 mmol, 1 eq.) and AIBN (1.45 mg, 0.009 mmol, 0.1 eq.) were dissolved in dioxane (10 mL). The flask was sealed with a rubber septum and then purged using argon for 15 min. The flask was heated at 60 °C for 16 h under magnetic stirrer. The reaction was stopped by exposing the solution in open air and the polymer/monomer mixture was precipitated in hexane (100 mL). The polymer R1 was obtained as a slight yellow powder with a yield of 44.3% (0.443 g) and it was characterized while using NMR spectroscopy and SEC.

#### 2.3.3. Determination of the Lower Critical Solution Temperature (LCST)

Homopolymer, PNIPAAm (5 mg) was dissolved in 5 mL of deionized water, 0.1 M NaSCN, and 0.1 M Na_2_SO_4_, respectively. The absorbance at 350 nm (A_350_) was recorded for temperatures that ranged from 25 to 45 °C. The experiment was done in duplicate.

#### 2.3.4. Preparation of PNIPAAm Gels via FRP

In a flat bottom glass vial, NIPAAm (0.2 g, 1.767 mmol), *N*,*N′*-methylenebis(acrylamide), and AIBN (0.3 mg, 0.002 mmol, 0.1% mol) were dissolved in dioxane. The flask was sealed with a rubber septum and then purged using argon for 15 min. The flask was heated at 60 °C for 16 h to ensure high conversion rates across the bulk volume of the samples. The gel was soaked in deionized water, which was replaced every 30 min. to remove the unreacted monomer and solvent; subsequently the gel was freeze-dried for 2 days. The amount of crosslinker and solvent were changed, and the different reaction conditions are reported in Table 1. Additionally, Fluorescein *O*-methacrylate (2 mg) or Fluorescein (2 mg) were incorporated respectively in the reaction mixture of F2 and the gels were used without any purification steps to establish their hydrolytic stability and release profile.

#### 2.3.5. Preparation of PNIPAAm Gels via RAFT Polymerization

In a flat bottom vial sample, NIPAAm (0.2 g, 1.767 mmol, 90 eq.) *N*,*N′*-Methylenebis(acrylamide), 2-(dodecylthiocarbonothioylthio)-2-methylpropionic acid (6.8 mg, 0.019 mmol, 1 eq.) and AIBN (0.3 mg, 0.002 mmol, 0.1 eq.) were dissolved in dioxane. The flask was sealed with a rubber septum and purged using argon for 15 min. The flask was heated at 60 °C for 16 h. The gel was soaked in deionized water which was replaced every 30 min. to remove unreacted monomer and solvent and the gel was freeze-dried for 2 days. The reaction conditions are reported in Table 1. Additionally, Fluorescein *O*-methacrylate (2 mg) and Fluorescein (2 mg) were incorporated respectively in the reaction mixture of R2 and the gels were used without any purification steps to establish their hydrolytic stability and release profile.

#### 2.3.6. Scanning Electron Microscopy (SEM)

A fragment of freeze-dried gel was attached to a self-adhesive carbon disc mounted on a 25 mm aluminium stub. The stub was coated with 25 nm of gold using a sputter coater. The stub was then placed into a FEI Quanta 200 FEG SEM for imaging at 5 kV accelerating voltage using secondary electron detection.

#### 2.3.7. Swelling Testing

Each freeze-dried gel was placed in 2 mL of deionized water, 0.1 M NaSCN and 0.1 M of Na_2_SO_4_ respectively at room temperature. The weight of each gel was recorded every 10 min. for the first hour and then every hour by collecting samples from their respective solutions followed by gentle wiping before weighing. After 24 h, the temperature was increased to 37 °C and the weight of the gel was recorded every 10 min. for the first hour and then every hour. The swelling ratio (SR) of the gels were determined by using the following Equation (1), where *m*_t_ is the mass of the swollen gel sample at time *t* and *m*_f_ is the mass of the freeze-dried gel sample. The SR represents the equilibrium weight change of PNIPAAm hydrogels during the immersion in the various solvents. The experiment was triplicated for each gel.

% SR = *m*_t_/*m*_f_ (1)

#### 2.3.8. Determination of the Volume Phase Transition Temperature (VPTT)

The freeze-dried gels G2F and G2R were each placed in 2 mL of deionized water, 0.1 M NaSCN, and 0.1 M of Na_2_SO_4_, respectively, at temperatures that ranged from 8 to 45 °C. After 24 h, the weight of each gel was measured at the different temperature. The VPTT was determined by using the evolution of the SR as a function of the temperature. The experiment was triplicated.

#### 2.3.9. Stability Study

The gels from the copolymerization with fluorescein *O*-methacrylate were left in water, 0.1 M NaSCN and 0.1 M Na_2_SO_4_ (10 mL), respectively. 1 mL aliquot was removed and immediately replaced with fresh solution to maintain the same volume at predetermined time intervals. The absorbance of each aliquot was recorded at 490 nm (A_490_) and the % of the fluorescein release was determined while using a calibration curve that defined the evolution of the A_490_ as a function of the concentration of fluorescein *O*-methacrylate. The experiment was triplicated.

#### 2.3.10. Release Study of Fluorescein 

Fluorescein-loaded gels were placed in a vial containing 10 mL of water, 0.1 M NaSCN, and 0.1 M Na_2_SO_4_, respectively. 1 mL aliquot was removed and then immediately replaced with fresh solution to maintain the same volume. The A_490 nm_ of the aliquot was read and the % of Fluorescein release was determined from a conventional curve that defines the evolution of the A_490 nm_ as a function of the concentration in Fluorescein. The experiment was triplicated.

## 3. Results and Discussion

### 3.1. Synthesis of Homopolymer PNIPAAm and Study of Its LCST

**Figure 1:**
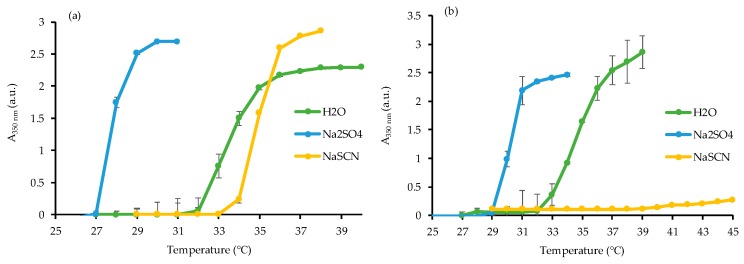
Absorbance (A_350_) as a function of temperature for (**a**) F1 and (**b**) R1 in deionized water, 0.1 M Na_2_SO_4_, and 0.1 M NaSCN solutions.

PNIPAAm homopolymer F1 and R1 were first synthesized while using conventional free radical and RAFT polymerization, respectively. For the RAFT made polymers, a sufficiently high degree of polymerization (DP) of 100 was chosen, so that the effect of CTA end-groups on the macroscopic properties could be virtually eliminated. The reaction was carried out in dioxane for 16 h at 60 °C while using AIBN as initiator, and a chain transfer agent (CTA) was added in the case of the RAFT polymerization while maintaining the same amount of AIBN. A monomer conversion of over 80% was determined while using NMR spectroscopy for both reactions. R1 and F1 PNIPAAm homopolymers were characterized while using NMR spectroscopy (Figure S1) and GPC (Figure S2) to confirm the molecular structure and the molecular weight. A Đ_M_ of 1.16 was found for R1 whereas the Đ_M_ value for F1 was 2.55. The narrow molecular weight distribution of R1 (i.e., Đ_M_ ≤ 1.2) confirmed the success of the RAFT polymerization of NIPAAm. Hence, these reaction conditions were satisfactory to be further used in the preparation of PNIPAAm gels. Additionally, the LCST of F1 and R1 were measured in deionized water, 0.1 M NaSCN, and 0.1 M Na_2_SO_4_ solutions to investigate the influence of kosmotrope and chaotrope solutes. More specifically, the LCST was determined as the intercept of the tangents to the two linear portions of the graph reporting the evolution of the absorbance at 350 nm (A_350_) as a function of temperature (Figure 1).

As expected, a similar LCST of 32 °C for PNIPAAm R1 and F1 in deionized water was found, which corroborates with the well-reported LCST of PNIPAAm in the literature [31,32]. The LCST of PNIPAAm decreased in a 0.1 M Na_2_SO_4_ solution (27 °C for F1 and 29 °C for R1). This is due to the fact that the H-bonding network between PNIPAAm and water molecules is disrupted via the presence of the anion SO_4_^2−^, which in turn results in the decrease of the LCST. On the other hand, the LCST of PNIPAAm increased in NaSCN to 33 °C for F1 and was nearly suppressed in the R1 sample, possibly due to the formation of low optical density coacervates. Unlike the anion SO_4_^2−^, the presence of SCN^−^ enhanced the hydrogen bonding network, which resulted in an increase of the LCST [30,33]. These results on the linear PNIPAAm homopolymers served as a baseline to perform a systematic study on similarly prepared gels in order to elucidate their behavior in the same solutions and the potential influence of the polymerization method for their preparation.

### 3.2. PNIPAAm Gels

#### 3.2.1. Preparation

The PNIPAAm gels were prepared while using conventional free radical (G1F–G4F) and RAFT polymerization (G1R–G4R). A crosslinker *N*,*N′*-Methylenebis(acrylamide) of different amount (i.e., 2.6%, 5%, and 10% mol for G1, G2/G4, and G3, respectively) was added to the reaction medium, as described for the homopolymerization of NIPAAm, however under static conditions (Table 1). For PNIPAAm gels G4, the copolymerization was carried out in twice as less medium to determine the influence of the monomer concentration on the gel properties. For each gel, the gel fraction was calculated and then found to be relatively high ranging from ~76% to 92%.

#### 3.2.2. SEM Imaging

**Figure 2:**
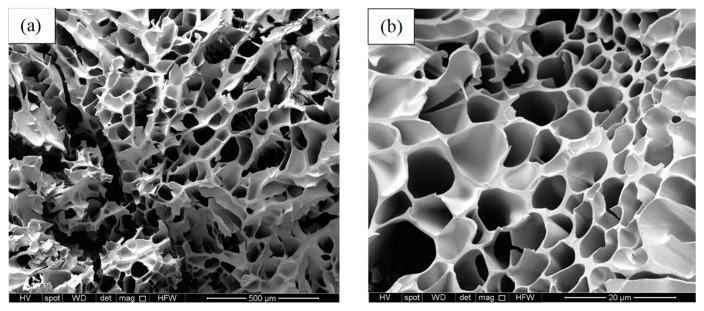
Scanning Electron Microscopy (SEM) images of G2F (**a**) and G2R (**b**).

The microstructure of the gels was investigated while using SEM and the representative images of G2F and G2R, respectively, are shown in Figure 2. Interestingly, we found that the RAFT made gels had more than 10-fold smaller pore size as compared to the FRP-made ones (6 ± 2 µm, and 67 ± 12 µm, for G2R and G2F, respectively) and they were more homogenous across the polymer network; this marked difference in the polymer network texture partly explains the significant differences in the swelling and release properties of these materials as the RAFT-gels have a larger interfacial area with their solute as per mass of polymer, and hence the overall swelling/de-swelling kinetics are more rapid and more pronounced, as we confirm in later experiments.

#### 3.2.3. Evaluation of Physical Properties

##### Swelling Study

The swelling/deswelling behavior of each gel was studied in different media. Initially, the gels were soaked in water to remove any residual monomer and solvent, and were then freeze-dried. Subsequently, each gel was immersed in the medium at room temperature and the weight was recorded over time. After 24 h, the temperature was increased to 37 °C, and the weight of each gel was again recorded over time.

• Influence of the amount of crosslinker

**Figure 3:**
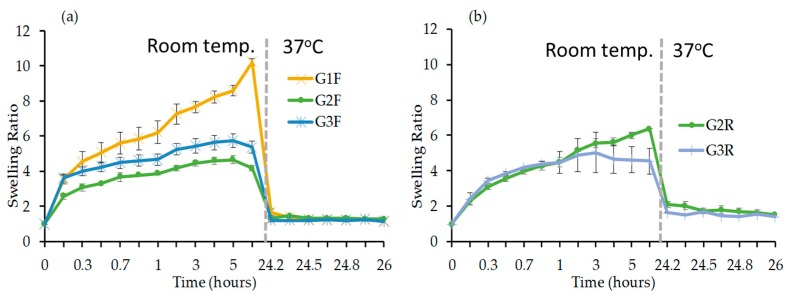
Evolution of the swelling ratio (SR) as a function of time in deionized water at room temperature for 24 h and then at 37 °C (shown as a grey line) for another 4 h for PNIPAAm gels prepared using (**a**) free radical polymerization (FRP) and (**b**) RAFT polymerization.

The SR was determined for PNIPAAm gels prepared while using a different amount of crosslinkers (Figure 3). The swelling profile rate of the FRP gels consists of an initial burst phase, followed by a sustained swelling (Figure 3a). For gel G1F (i.e., 2.5 mol.% of crosslinker), the SR reached ~10.1 after 24 h at room temperature, whereas the ratio of G2F and G3F decreased by a factor ~2 (i.e., 4.7 and 5.7, respectively), due to the higher amount of crosslinker that renders the polymer network much denser when compared to that of GF1. Furthermore, the SR of the gels that were prepared using RAFT polymerization with 5 (G2R) and 10% mol. (G3R) of crosslinker were found to be 6.4 and 5.0, respectively (Figure 3b), hence demonstrating the dependence of the SR with the amount of crosslinker again. To be noted, the SR of G1R could be not evaluated because of the lack of gel consistency; we believe that this constitutes indirect evidence that non-defined chemical entanglement from side reactions (i.e., chain-chain crosslinking) contribute to the formation of gels by FRP; this explains the inability of RAFT to form gels at lower crosslinker feeds, simply because the reaction mechanism is more well-defined with a minimization of side-reactions [34] leading to better defined crosslinks overall and this is also corroborated by the difference between the SR of FRP and RAFT gels. RAFT polymerization allows for the incorporation of chemical crosslinks in a highly controlled manner, hence forming a well-defined hydrogel network [21,22,23,24,25,26]. Finally, after 24 h, the temperature was increased to 37 °C, and the SR of each gel instantly dropped to 1 due to the loss of PNIPAAm solubility in water above 32 °C.

• Influence of the monomer concentration

**Figure 4:**
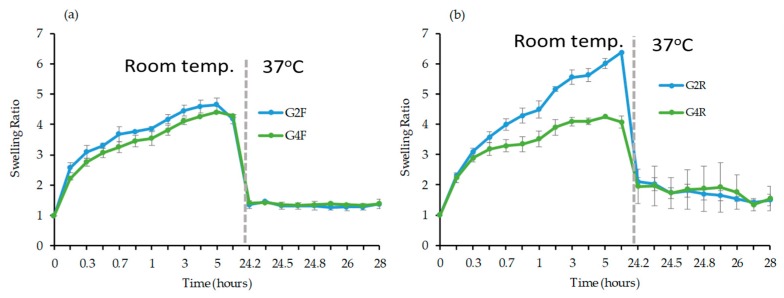
Evolution of the SR as a function of time in deionized water at room temperature for the first 24 h and then at 37 °C (shown as a grey line) for another 4 h for PNIPAAm gels (**a**) G2F and G4F and (**b**) G2R and G4R.

The gels G4F and G4R were prepared while using 5 mol.% of crosslinker in a two-fold more concentrated reaction medium than G2 in order to investigate the influence of the monomer concentration on the swelling properties of the gels. Again, a sustained swelling rate following an initial burst phase was observed for both G4F and G4R (Figure 4). The SR of G4F gel prepared while using FRP (Figure 4a) was found to be 4.4 and it remained similar to G2F with a SR value of 4.7, hence indicating the virtually complete absence of influence of the monomer concentration. Remarkably, a significant decrease of the SR was observed for the G4R when compared to G2R where the SRs were 4.1 and 6.4, respectively (Figure 4b). The increase of monomer concentration must have increased the rate of the polymerization, probably resulting in high monomer conversion under the tested reaction conditions that could cause the loss of the control of the RAFT polymerization, which in turn could lead to inconsistent incorporation of crosslinks within the network [35,36,37]. To be noted, the increase of the temperature to 37 °C after 24 h resulted in the loss of the total water uptake of the gels.

• Influence of the nature of the swelling medium

**Figure 5:**
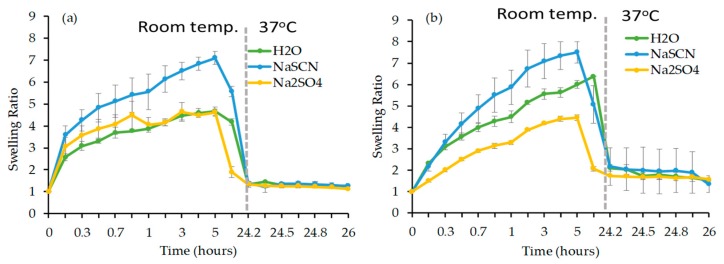
The SR as a function of time in deionized water, 0.1 M NaSCN and 0.1 M Na_2_SO_4_ solution respectively at room temperature for the first 24 h and then 37 °C (shown as a grey line) for another 4 h for (**a**) G2F and (**b**) G2R gels.

The gel samples G2F and G2R were shortlisted to investigate the influence of a strong kosmotrope and chaotrope solute on the swelling properties. Figure 5 displays the SR of G2F and G2R as a function of time in water, 0.1 M Na_2_SO_4_ and 0.1 M NaSCN solution, respectively. In the case of G2F, the SR increased by a factor 2 in NaSCN solution, whereas the SR in Na_2_SO_4_ solution was similar to that in water (i.e., 4). Furthermore, the swelling profile of G2F in NaSCN and Na_2_SO_4_ solutions were similar to that in water. On the other hand, the SR of G2R increased in NaSCN solution (i.e., ~8), but decreased in the Na_2_SO_4_ solution (i.e., ~4) when compared to that in water (i.e., ~6). The swelling profile seems to follow a pseudo-zero order in NaSCN and Na_2_SO_4_, while an initial burst swelling phase was observed in water, followed by a sustained swelling for G2R.

The difference of swelling rates in each medium was in line with the LCST values of the homopolymers that were synthesized by RAFT polymerization and FRP. As expected, the LCST increased in the chaotrope solute and decreased in the kosmotrope solute, which was also reflected in the swelling behavior of the gels. This set of experiments shows a clear difference of the behavior of the gels that is based on the method of polymerization and provides important insight on the role of the molecular structure to the macroscopic properties of the final material.

• Influence of the temperature in VPTT determination

**Figure 6:**
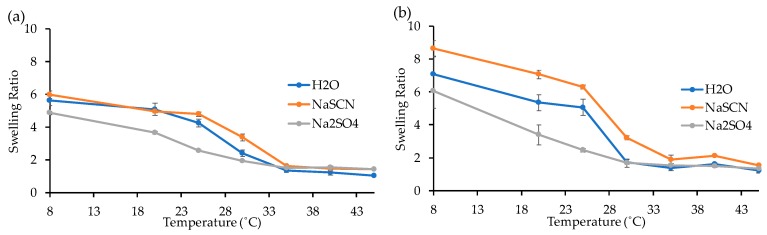
Evolution of the SR as function of the temperature for gel G2F (**a**) and G2R (**b**).

The gels G2R and G2F were used to investigate the influence of temperature on their swelling properties in distilled water, NaSCN and Na_2_SO_4_ solution, respectively. The SR of each gel was measured at different temperatures, as shown in Figure 6. In both cases, the SR decreases with the increase of the temperature with a higher decrease slope around the LCST onset for both samples. At 35 °C, the SR of the gels G2F (Figure 6a) immersed in water, NaSCN and Na_2_SO_4_ solution respectively was found to be ~1, indicating the complete loss of the water uptake. At 8 °C, a SR of 4.9, 5.6 and 6.0 for G2F was found in Na_2_SO_4_ solution, water and NaSCN solution, respectively (Figure 6a). G2R had considerably higher SR values at 8 °C, namely, 6.1, 7.0, and 8.7, at Na_2_SO_4_, NaSCN, and H_2_O, respectively (Figure 6b).

##### Dye Release Study

**Figure 7:**
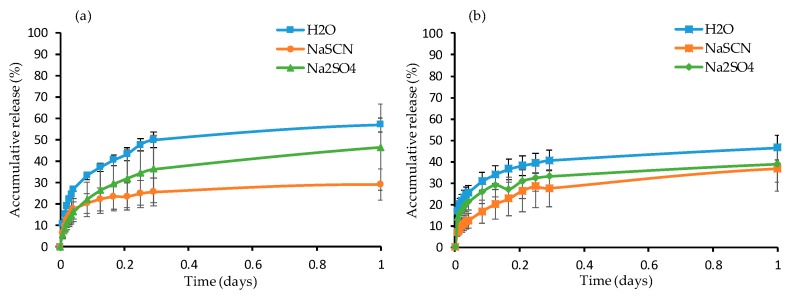

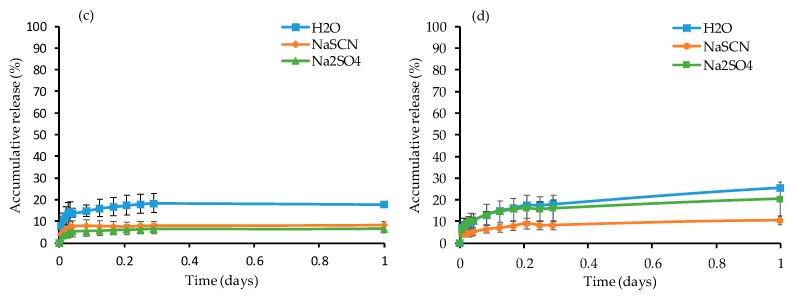
Accumulative release of fluorescein as a function of time from G2F at (**a**) room temperature and (**b**) 37 °C and from G2R at (**c**) room temperature and (**d**) 37 °C in water, 0.1 M NaSCN, and 0.1 M Na_2_SO_4_ solution, respectively.

Another set of gels was synthesized with 5 mol.% of crosslinker in 1 mL of solvent while using FRP and RAFT polymerizations, in which 2 wt.% of fluorescein was loaded. The gels were immersed in water, 0.1 M NaSCN, and 0.1 M Na_2_SO_4_ solution at room temperature and 37 °C. An aliquot was collected every 10 min. during the first hour, followed by aliquot collection every hour and were analyzed while using UV-vis spectroscopy to measure the release (Figure 7).

For all gels, the release profile rate was sustained following an initial burst release phase. The FRP gels had higher release rate in water and Na_2_SO_4_ solution at room temperature (Figure 7a) than 37 °C (Figure 7b) due to the loss of PNIPAAm solubility above 27 °C (in Na_2_SO_4_) and 32 °C (in water). More specifically, 56.9% and 46.4% of fluorescein was released in water and Na_2_SO_4_ solute, respectively, at room temperature after one day while 46.5% and 38.9% was released at 37 °C. However, the release rate of fluorescein was higher in the NaSCN solution at 37 °C than at room temperature and this can be attributed to the increase of the LCST of PNIPAAm in NaSCN as previously shown; in fact, 36.1% and 29.0% of fluorescein was released in NaSCN at 37 °C and room temperature, respectively. For the RAFT gels (Figure 7c,d), the release rate in each medium was higher at 37 °C than room temperature. Only 17.9%, 6.6% and 8.3% of fluorescein was released in water, Na_2_SO_4_, and NaSCN at room temperature (Figure 7c) after one day, whereas the release was found to be 25.6%, 20.4%, and 10.4%, respectively, at 37 °C (Figure 7d). The increase can be due to the gel contraction, which presumably enhances the fluorescein release in the solute above the LCST of PNIPAAm.

It is apparent that the release rate from the RAFT gels was lower when compared to that of the FRP gels independently of the temperature and the release medium, and this can be explained via the distribution and size of the polymer mesh network. RAFT polymerization allows for well-distribution of the crosslinks in a highly ordered and homogeneous manner, and hence it is expected to form gels with well-defined polymer mesh and pore distribution, as also seen in SEM images (Figure 2). However, the crosslinks in the FRP gels are expected to be more randomly distributed, given the non-controlled polymerization rate resulting in irregular polymer mesh and possibly broader pore size distribution that macroscopically led to higher release rates overall.

Intriguingly, the FRP gels also showed reduced release rates above the LCST as compared to the RAFT-gels; we attribute this to the differences in pore size and the more ill-defined polymer network, which results in less interfacial interactions with the aqueous solute (FRP gels had 10-fold larger pores and hence less polymer-water interfacial area to augment release events) and the possible increase of hydrophobic interactions of the sparingly-soluble fluorescein molecules with the hydrophobized polymer mesh above the LCST.

Again, it is noteworthy to mention the marked differences on the release profiles that are based on the different polymerization routes; given that these materials are routinely used as drug release matrices, it is critical to know the exact molecular parameters that govern the behavior of these materials.

##### Hydrolytic Stability of Co-Monomers

**Figure 8:**
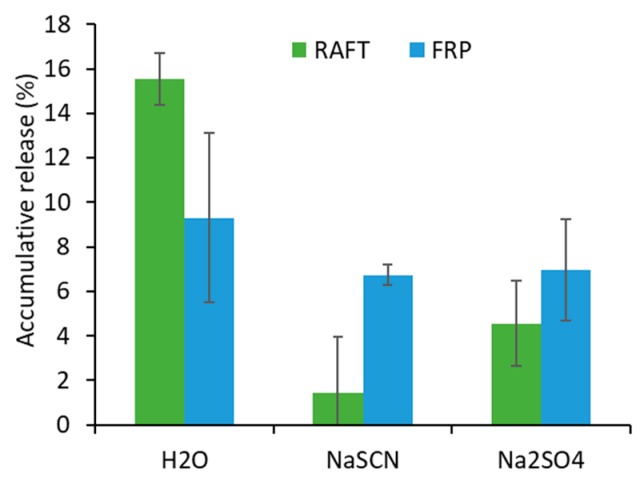
Accumulative release of polymer-bound fluorescein from G2R and G2F after four days in deionized water, 0.1 M NaSCN, and 0.1 M Na_2_SO_4_ solutions, respectively.

Another set of PNIPAAm gels was prepared with the addition of 2% of the co-monomer, fluorescein *O*-methacrylate. The hydrolytic stability was assessed while measuring the release of fluorescein, which was covalently attached to the gel network directly on the polymers’ backbone. The gels were placed in water, 0.1 M NaSCN, and 0.1 M Na_2_SO_4_ solution, respectively, at 37 °C. The release of fluorescein was analyzed over time while using UV-vis spectroscopy (Figure S3). After four days, each solution was analyzed while using UV-vis spectroscopy to determine the release of fluorescein as an indicator of the chemical stability of the polymer network (Figure 8).

The percentile release of fluorescein from the FRP gels was found to be 9.3%, 6.7%, and 6.9% in water, NaSCN, and Na_2_SO_4_ solutions, respectively after four days. However, the differences were not statistically significant indicating no influence of the medium on the hydrolytic stability of the FRP gels. On the other hand, 15.5%, 1.4%, and 4.6% of fluorescein was released after four days in water, NaSCN, and Na_2_SO_4_ solutions, respectively, from the RAFT gels. The release of fluorescein was significantly enhanced in water when compared to that in NaSCN and Na_2_SO_4_, although the difference of release was not significant in NaSCN and Na_2_SO_4_. The RAFT gels were hydrolytically more stable in both NaSCN and Na_2_SO_4_ solution than in water. When comparing the gels’ stability as a function of the polymerization mechanism, we found that the RAFT gels were less stable in water but more stable in the NaSCN solution as compared to FRP gels, while a similar stability in Na_2_SO_4_ solution was highlighted. It should be noted that the fluorescein molecule is linked via an ester bond on the polymer backbone, which is hydrolytically less stable than the amide NIPAAm monomer units and the crosslinker moieties. Therefore, this study does not reflect the actual hydrolytic stability of the polymer network, but rather gives an indication of the capacity of the network to retain pendant units from co-monomer synthons, which is a common synthetic strategy in hydrogels’ design—that is, NIPAAm is often combined with other co-monomers to incorporate additional functionalities on the final product.

## 4. Conclusions

To summarize, the PNIPAAm gels were successfully prepared with different amounts of crosslinker and monomer concentration while using RAFT polymerization and FRP. It was shown by SEM imaging that the two methods of polymerization produce polymer networks of different texture and microstructure, which in turn determine their macroscopic properties, such as swelling, volume phase transition temperature, dye release, and hydrolytic stability. The swelling, release, and stability properties of the PNIPAAm gels were investigated in water, and in a strong komostrope and chaotrope solute. Independently of the polymerization mechanism, the SR decreased with the increase of concentration of monomer feed in the polymerization reaction and amount of crosslinker, respectively. Furthermore, the SR was higher in NaSCN and lower in Na_2_SO_4_ solution compared to that in water due to the capacity of each solute to either promote or disrupt the hydrogen bonding with water molecules. Interestingly, the swelling profile of the RAFT and FRP made gels was markedly different, which indicated the influence of the polymerization mechanism on the hydrogels macroscopic properties. Additionally, the FRP gels showed higher release profile rates when compared to the RAFT gels. Finally, the hydrolytic stability behavior of RAFT prepared gels differed from FRP gels mainly due to the difference in the gel homogeneity. Overall, these results indicate that the method of preparation is a critical factor in the molecular properties of PNIPAAm gels and their macroscopic properties, and hence careful considerations should be taken in the design of these materials, especially when already published protocols from the literature are adopted.

## Figures and Tables

**Table 1:** Reaction conditions for the preparation of Poly(*N*-isopropylacrylamide) (PNIPAAm) gels via free radical and reversible addition-fragmentation chain transfer (RAFT) polymerization.

Gel_ID	Crosslinker			*V*_dioxane_ (mL)	Monomer Concentration (M)	Gel Fraction (%)	
	mg	mmol	%mol			FRP	RAFT
G1	7.2	0.047	2.6	1	1.814	92.0	–
G2	14.3	0.093	5	1	2.697	79.2	76.3
G3	28.7	0.186	10	1	1.953	79.2	76.3
G4	14.3	0.093	5	0.5	3.720	82.9	88.5

## Supplementary Materials

The following are available online at https://www.mdpi.com/1996-1944/12/17/2697/s1, Figure S1: ^1^H NMR (400 MHz, CDCl_3_) of PNIPAAm synthesised using (a) FRP and (b) RAFT polymerization, Figure S2: GPC trace of PNIPAAm synthesised using FRP (green) and RAFT polymerization (blue), Figure S3: Release of fluorscein from G2F (a) and G2R (b) in different media.
