# Supplementary material for: Comparison of Thermoresponsive Hydrogels Synthesized by Conventional Free Radical and RAFT Polymerization
Source: Materials (Basel). 2019 Aug 23;12(17):2697. doi: 10.3390/ma12172697 (PMC6747592; doi:10.3390/ma12172697)
Supplement: Supplementary file 1 [file materials-12-02697-s001.zip › materials-467469-supplementary.pdf]

materials

Supporting Information

Comparison of Thermoresponsive Hydrogels Synthesized by Conventional Free Radical and RAFT polymerization

Fanny Joubert * Peyton Cheong Phey Denn, Yujie Guo, and George Pasparakis *

UCL School of Pharmacy, 29-39 Brunswick Square, London WC1N 1AX, UK
* Correspondence: f.joubert@ucl.ac.uk (F.J.); g.pasparakis@ucl.ac.uk (G.P.)

Received: 4 March 2019; Accepted: 20 August 2019; Published: date

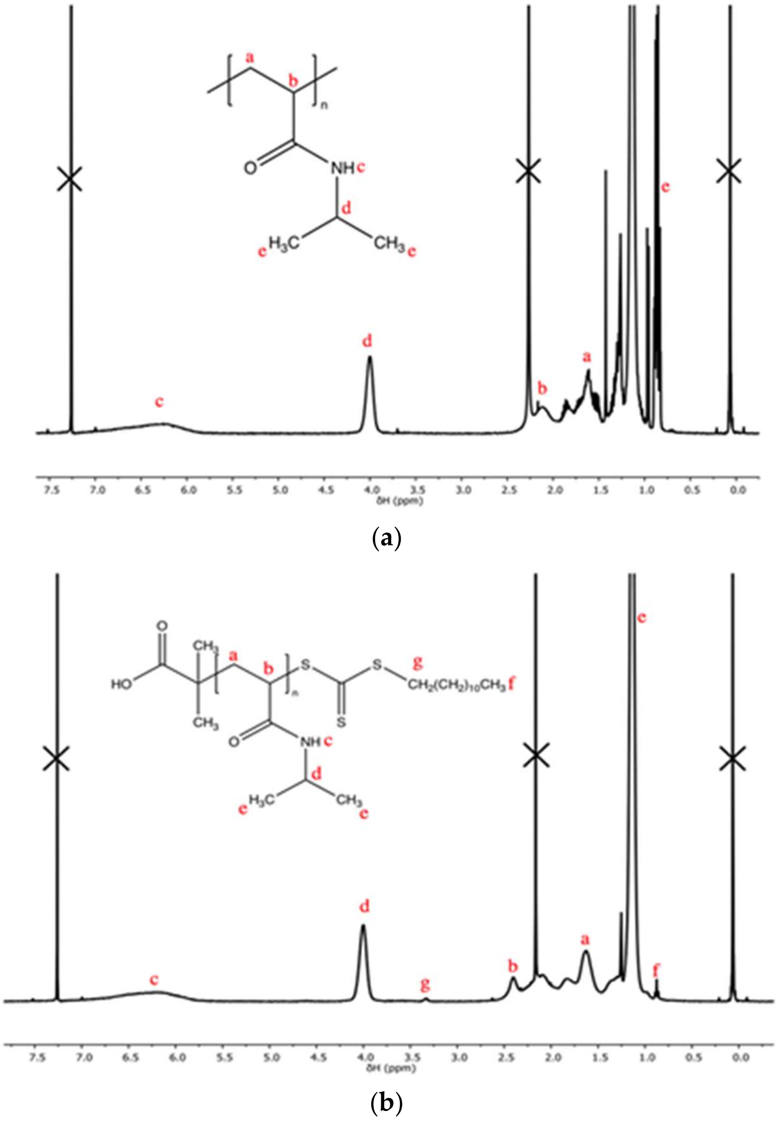

Figure S1. ^{1}H NMR (400 MHz, $CDCl_3$) of PNIPAAm synthesised using (**a**) FRP and (**b**) RAFT polymerization.

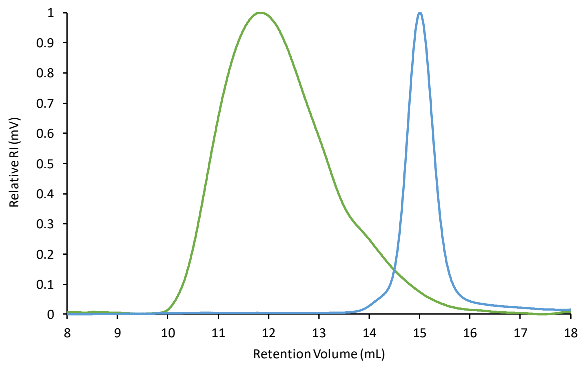

Figure S2. GPC trace of PNIPAAm synthesised using FRP (green) and RAFT polymerization (blue).

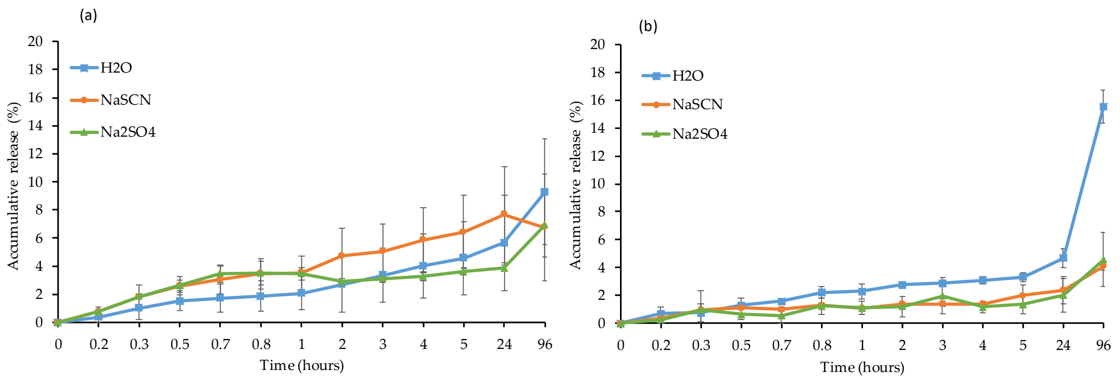

Figure S3. Release of fluorscein from G2F (**a**) and G2R (**b**) in different media.
